# Concurrent Primary Follicular Lymphoma and a Mature Cystic Teratoma of the Ovary: A Case Report and Review of Literature

**DOI:** 10.1155/2022/5896696

**Published:** 2022-02-25

**Authors:** Sakara Hutspardol, Yi Li, Valerie Dube, Jan Delabie

**Affiliations:** ^1^Department of Pathology, Vancouver General Hospital, Vancouver, British Columbia, Canada; ^2^Department of Pathology and Laboratory Medicine, University of British Columbia, Vancouver, British Columbia, Canada; ^3^Trillium Health Partners/Credit Valley Hospital, Mississauga, Ontario, Canada; ^4^Department of Laboratory Medicine and Pathobiology, University of Toronto, Toronto, Ontario, Canada; ^5^Laboratory Medicine Program, University Health Network and University of Toronto, Toronto, Ontario, Canada

## Abstract

Primary lymphoma concurrent with teratoma of the ovary is exceedingly rare. Based on our review of the literature, there are only 8 case reports describing concurrent primary diffuse large B-cell lymphoma and teratoma. Here, we report the first case of primary follicular lymphoma concurrent with mature ovarian cystic teratoma, which, to our knowledge, has not been described in the literature.

## 1. Introduction

Mature cystic teratomas are the commonest ovarian tumor, accounting for 20% of all ovarian neoplasms and up to 60% of benign ovarian tumors during the reproductive years [1]. However, some tumors are not detected until postmenopause [2].

Most ovarian lymphomas represent secondary involvement by a systemic disease [3]. In comparison, primary ovarian lymphomas are rare and account for less than 1% of all cases of non-Hodgkin's lymphomas (NHLs) [4]. Concurrent involvement by NHL and MCT is exceedingly rare with only eight reported cases in the literature (Table 1). In all cases, diffuse large B-cell lymphoma was the lymphoma type.

Here, we report the occurrence of primary Stage IE, Grade 3A ovarian follicular lymphoma within a MCT in a 62-year-old woman who underwent a laparoscopic bilateral salpingo-oophorectomy and omentectomy. Thorough radiologic investigations and staging bone marrow studies excluded a systemic disease. To our knowledge, this is the first case report of primary follicular lymphoma concurrent with MCT. The exclusive presence of follicular lymphoma within the MCT suggests it may have arisen from it.

## 2. Case Report

A 62-year-old female presented with pelvic discomfort without constitutional symptoms. CT abdomen demonstrated a 15.1 × 12.2 × 16.3 cm right ovarian solid and cystic mass with minimal calcification. Laboratory investigation reveals minimally elevated CA-125 of 55 (normal < 35 U/mL) and CA 19-9 less than 3 (normal < 27 kU/L). Physical examination showed a palpable right lower quadrant abdominal mass without lymphadenopathy.

The patient underwent a laparoscopic bilateral salpingo-oophorectomy, omentectomy, and right ureterolysis. A 15 cm diameter right solid adnexal mass attached to the pelvic wall was excised with rupture of a cystic component.

Histological examination revealed fragments of benign squamous epithelium, ciliated respiratory-type epithelium, and hair component-associated foreign-body type granulomatous reaction. There were also cystic areas which lined by the squamous epithelium. The overall appearance was consistent with a mature cystic teratoma.

**Figure 1:**
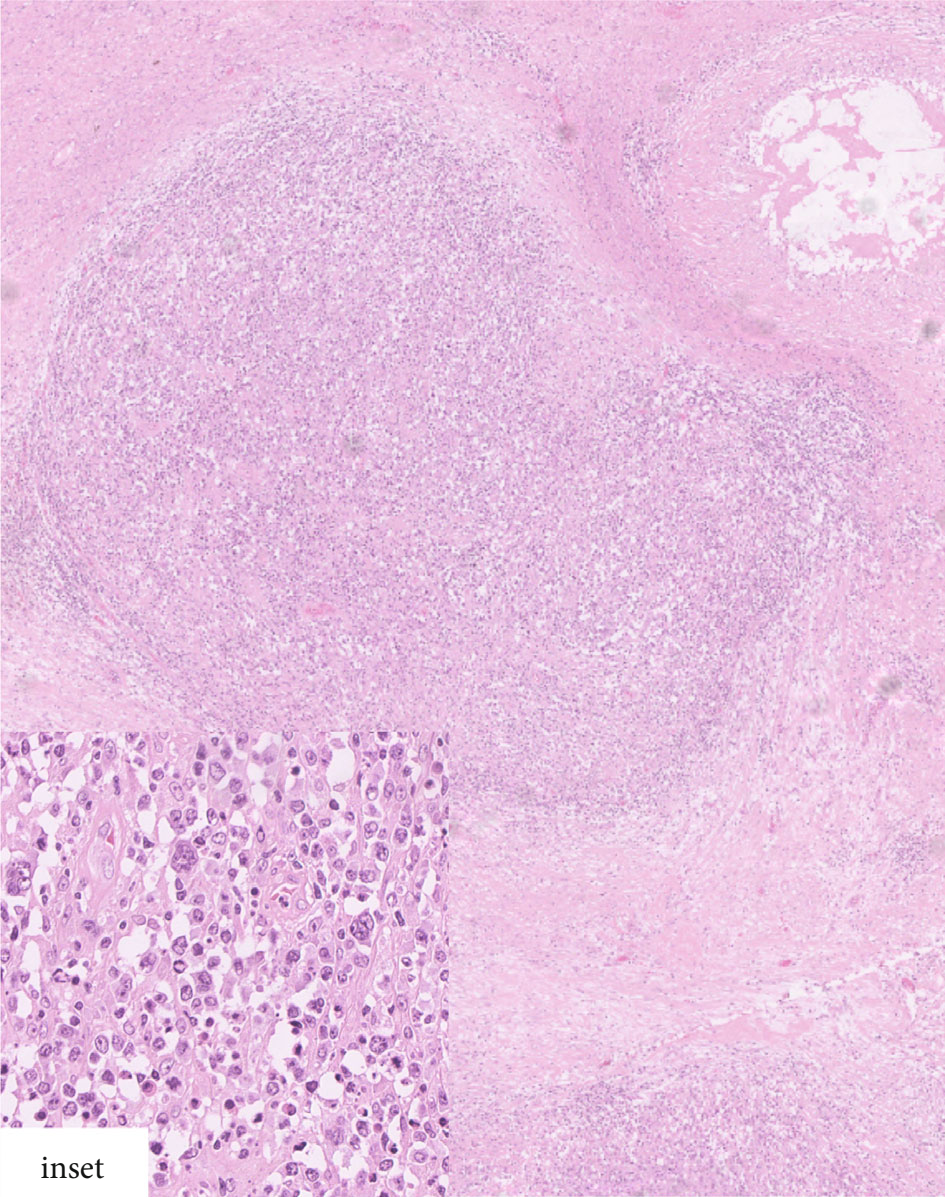
Hematoxylin and eosin staining (H & E 200x). Composite histology of follicular lymphoma within the mature cystic teratoma. The inset shows high-power view of the follicular lymphoma component comprising predominantly centroblasts and scattered centrocytes (H & E 400x).

Further microscopic examination revealed very focal nodular infiltrates of atypical lymphoid cells within the teratoma (Figure 1). These lymphoid aggregates were composed primarily of intermediate to large-sized atypical cells with irregular nuclei, open chromatin, distinct nucleoli, and abundant cytoplasm, morphologically consistent with predominantly atypical small and large centroblasts (more than 15 per high power field). Scattered small centrocytes were also seen. Focal necrosis was seen in some of the aggregates.

**Figure 2:**
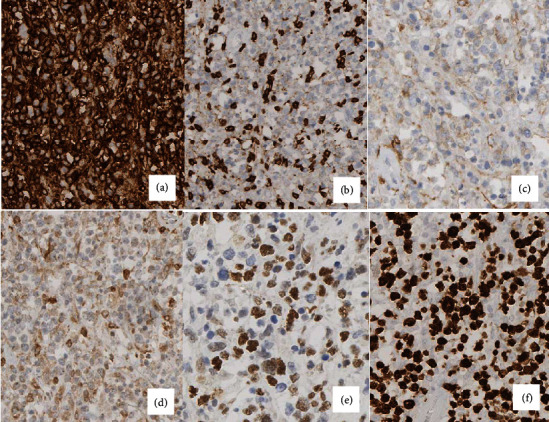
Immunohistochemical stains (400x) show that the atypical lymphocytes are positive for CD20 (a), negative for CD3 (b), weakly positive for CD10 (c), weakly positive for BCL2 (d), and positive for BCL 6 (e), with increased Ki-67 proliferation index at 80% (f).

Immunoperoxidase studies showed that the atypical cells are B lymphocytes with germinal center cell immunophenotype (expressing CD10 and BCL6) and showed aberrant expression of BCL2 (weak). They are associated CD21-positive follicular dendritic cell meshworks. The Ki-67 proliferation index is 80% in the neoplastic cells. The overall morphologic and immunohistochemical findings are consistent with a grade 3A follicular lymphoma (Figures 2(a)–2(f)).

The patient underwent further staging investigations, including a whole-body PET scan and CT scans of the head and neck, chest, abdomen, and pelvis, which show no evidence of systemic lymphoma. A bilateral bone marrow biopsy showed no bone marrow involvement. Therefore, the follicular lymphoma is primary to the ovary and arises as a part of the mature teratoma.

The patient was followed postoperatively every 3 months without further treatment and remained in complete clinical and radiological remission 18 months after the diagnosis.

## 3. Discussion

Mature cystic teratoma (MCT) is the most common germ cell neoplasm of the ovary in females of reproductive age. The majority of cases, particularly in younger women, are benign and treated with resection alone. In contrast, peri- and postmenopausal women with MCT have a higher risk of malignant transformation at approximately 1–2% with squamous cell carcinoma being the most common type of malignancy accounting for 80-90% of cases [5].

Primary lymphoma as a neoplastic component of ovarian teratoma is exceptionally rare with only 8 reported cases in the literature (Table 1); the lymphoma type reported in all cases is diffuse large B-cell lymphoma. Of the eight patients, five presented with localized unilateral disease, and one presented with bilateral ovarian involvement. The remaining two patients do not have complete staging investigation [6, 7]. Three patients were given R-CHOP chemotherapy and one given cyclophosphamide, mitoxantrone, and dexamethasone. All four patients who received postoperative adjuvant chemotherapy showed no evidence of disease recurrence upon follow-up [8–11]. Two patients underwent oophorectomy alone without any adjuvant chemotherapy but remained in complete remission [12]. The durations of postoperative follow-up ranged from 4 months to 14 months. The remainder two patients do not have any follow-up information [13].

The key features of concurrent primary ovarian lymphoma and MCT include a wide age range at diagnosis (ranging from 24 years to 68 year of age), minimal involvement by the lymphoid infiltrate, and localized disease (Table 1). Although the prognosis is excellent, the number of cases does not allow to comment on best treatment with respect to simple resection or resection with postoperative adjuvant chemotherapy. In addition, most cases have a short follow-up duration, ranging from 4 to 14 months and, as such, may not reflect the true clinical progression of the disease. Irrespectively, primary ovarian NHL has an excellent prognosis, particularly when diagnosed at early stage [4].

The exclusive presence of follicular lymphoma within the teratoma suggests it may have arisen from it. Hematolymphoid disease has previously been demonstrated to occur within germ cell tumors, especially in the mediastinum. Both myeloid neoplasia and histiocytic sarcoma have most frequently been demonstrated [14]. The detection of immature myeloid precursors in the germ cell tumor has been suggested as the cells of origin for these neoplasms [15].

## Figures and Tables

**Table 1:** Summary of previously reported cases of primary lymphoma of the ovary which coexists with mature cystic teratoma.

Author	Diagnosis	Age (years)	Side	Stage	Therapy	Follow-up (months)	Outcome
Seifer et al. (1986)	DLBCL	24	Right	Not available	Resection	Not available	Not available
McKelvey et al. (2003)	DLBCL, GCB subtype	52	Bilateral ovarian involvement by DLBCL, right with teratoma and left without teratoma	I_E_	Resection and 6 cycles of CMD	10	Complete remission
Maguire et al. (2015)	DLBCL, GCB subtype	68	Left	I_E_	Resection and 6 cycles of R-CHOP	14	Complete remission
Gandhi et al. (2012)	DLBCL, GCB subtype	36	Right	I_E_	Resection and 4 cycles if R-CHOP	4	Complete remission
Valli et al. (2014)	DLBCL associated with chronic inflammation	56	Right	I_E_	Resection and 6 cycles of R-CHOP	8	Complete remission
Bonneville et al. (2017)	DLBCL	24	Right	I_E_	Resection	6	Complete remission
Yorita et al. (2019)	Fibrin-associated DLBCL	57	Right	I_E_	Resection	4	Complete remission
Afzal et al. (2017)	High-grade B-cell lymphoma	45	Right	Not available	Not available	Not available	Not available

Abbreviations: DLBCL: diffuse large B-cell lymphoma; GCB: germinal center-B; CMD: cyclophosphamide, mitoxantrone, dexamethasone; R-CHOP: rituximab, cyclophosphamide, doxorubicin, vincristine, prednisone.
